# Exploring the valorization of green leaves accumulated as agricultural waste into plant-based fermented juices

**DOI:** 10.3389/fnut.2026.1823095

**Published:** 2026-04-21

**Authors:** Emine Gizem Acar, Dilara Devecioglu, Gulay Ozkan, Funda Karbancioglu-Guler, Derya Kahveci

**Affiliations:** Department of Food Engineering, Faculty of Chemical and Metallurgical Engineering, Istanbul Technical University, Istanbul, Türkiye

**Keywords:** agricultural waste, green leaves, upcycled beverage, water kefir fermentation, phenolic compounds

## Abstract

Every year, tons of green leaves generate in agricultural fields as waste after harvesting. This study aimed to explore the bioactivity of green leaves, and to evaluate their potential for upcycling into plant-based fermented beverage. Juices of broccoli, cauliflower, beetroot and black carrot leaves were obtained by cold pressing. The highest juice yield, total phenolic content (TPC), total flavonoid content (TFC), antioxidant capacity, and the least cytotoxicity were observed for beetroot leaves. While the *in vitro* bioaccessibility of TPC and TFC of juices varied between 21.34–33.30% and 21.34–30.95%, respectively, the recovery of their antioxidant capacity after the intestinal phase were higher, up to 98%. Three of the leaf juices (cauliflower, beetroot and black carrot) were fermented using water kefir for 48 h, and each juice had lactic acid bacteria and yeast count higher than 6 log CFU/mL. Fermented beetroot leaf juice was chosen for storage and sensory analysis due to the highest probiotic viability and sulforaphane concentration. The microbial growth, organic acid profile and physicochemical changes showed that the fermentation continued slowly during storage at 4 °C. Over a 21-day storage period, overall acceptability was found to be above average by untrained panelists. By this study, a step was taken to close the gap in the literature regarding the bioactivity and cytotoxicity of green juices, and a new perspective was reflected for the valorization of discarded leaves, paving the way for producing plant-based, fermented and upcycled beverages that are consumer acceptable and bioactive.

## Graphical abstract

**Figure:**
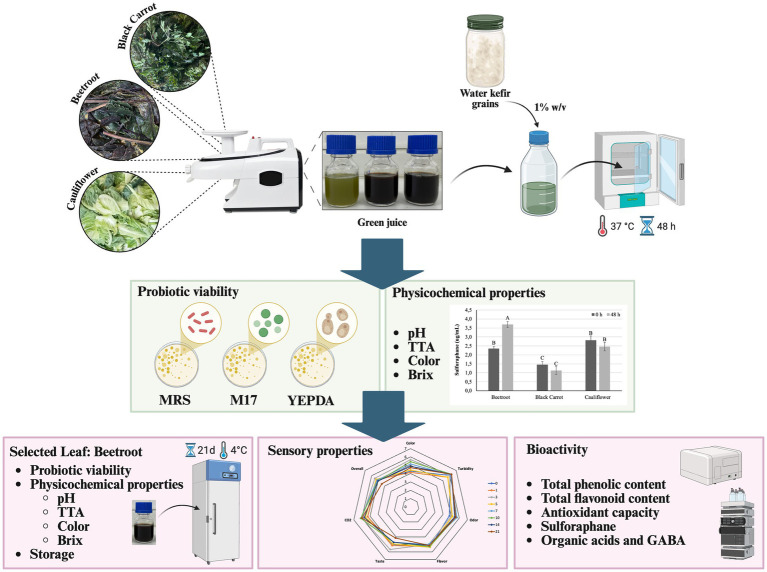

## 1 Introduction

The Food and Agriculture Organization (1) estimates that around one-third of food produced for human consumption globally is either lost or wasted. Preventing and reducing food loss and waste (FLW) is of great importance, since the global population is expected to reach approximately 10 billion by 2050 (2), which would correspond to an increase of about 60% in food demand. The food industry is now prioritizing the development of sustainable value chains, while consumers are increasingly seeking foods with positive health effects. An emerging approach in FLW reduction strategies has been valorization of food waste by extracting its functional and bioactive components. Application of food waste-derived components as food ingredients/products, so-called “upcycled ingredients/foods” (3), has been a global trend on the rise, which is also described as the best option to reduce FLW, together with donation, in Wasted Food Scale by EPA (4). Many researchers as well as industrial actors have been putting increasing effort into realizing upcycled food products, which has mainly been hindered due to legal regulations as well as consumer acceptance.

Production of agricultural commodities results in a vast amount of waste, mostly at the harvesting stage. Stems, leaves and stalks of vegetables are generally left on the field, or only used for animal feed production. Such practices are among the reasons why food production is responsible for one-fourth of global greenhouse gas emissions (5). Several attempts have been reported to valorize green leaves, main agricultural waste of vegetable production. *Brassicaceae* family, with members including cabbage, broccoli, cauliflower, and brussels sprouts, especially gained attention (6, 7) due to the high volume of production globally [over 186 million tons in 2022 (8)] as well as waste-to-product ratio; edible portion of broccoli and cauliflower, termed as florets, consist to only about 30–40% of the plant. Broccoli waste extracts were reported to have potential toward management of Alzheimer’s disease and aging (9), gentamicin-induced liver and kidney toxicity (10), cytotoxicity and antibacterial activity (11, 12), obesity and glucose intolerance (13). Cauliflower has also been subject to attempts in order to provide bioactive extracts from its green leaves (14–16). Root vegetables, on the other hand, produce lower amount of waste in the form of stems and leaves, when compared to *Brassicaceae* family; however, their widespread production globally amounts to a significant waste accumulation as well. Within this group, beetroot (17–19) and carrot (20–22) leaves have been used to produce bioactive extracts.

Valorization of agricultural waste to produce extracts enriched in bioactive compounds, especially phenolic group, can be tedious in terms of additional processes required. Existing reports relied on air (23), oven (12) or freeze (24) drying of the leaves prior to extraction. Additionally, many researchers [such as Liu et al. (25)] fractionated waste into stems and leaves, which is a labor-intensive step. Besides conventional solvent extraction (26), researchers applied microwave (14, 27), ultrasound (28), bacterial (15) or enzyme (16) assisted extraction, supercritical CO_2_ (29, 30), pressurized liquid (31), or subcritical (32) extraction. However, industrial scale valorization facilities require simpler systems, as several green biomass processing facilities focusing on similar raw materials demonstrate already (33, 34).

Production of leaf juice (LJ) from wasted leaves by cold-pressing, and utilizing it for human consumption would contribute to the reduction of FLW and upcycling efforts. Broccoli leaves were shown to have higher bioactivity when compared to other waste fractions (stems and inflorescences) of the plant (26), as well as the edible florets (25). Given their high bioactivity, incorporating these bioactive-rich leaves into the functional food market would also create diversity in meeting changing consumer demands. Additionally, fermenting LJs may be another option, since fermentation enhances the digestibility, nutritional quality, sensory attributes and shelf life, together with increasing the antioxidant and anticancer properties of juices (35). Water kefir, a complex polysaccharide matrix whose grains consist primarily of levan and dextran and support a symbiotic microbial community, is distinguished among fermented beverages for being traditional and versatile (36). Although the microbiota may vary depending on the origin and growth conditions, grains generally consist of yeasts, acetic acid and lactic acid bacteria (LAB), including *Lactobacillus* sp., *Lactococcus* sp., *Leuconostoc* sp. and *Streptococcus* sp. (37). Water kefir fermentation provides a nutritious option for a broader spectrum of consumers, including those preferring vegan and lactose-free diets (38), and imparts bioactive, anti-inflammatory, antimicrobial, antioxidant, and probiotic properties to the beverage (39). It also provides an unique aroma due to the metabolites released during fermentation (40), where the sensory properties depend on the culture, process conditions and beverage used as substrate (41).

The aim of this work was exploring the valorization of green leaves obtained from production of four vegetables (beetroot, black carrot, broccoli, cauliflower) with significant production volumes, and producing plant-based fermented juices. In an attempt to develop a sustainable and feasible value chain, the leaves were used as they are, without any pretreatments or extraction except juicing. The bioactivity and cytotoxicity of LJs, and the effect of water kefir fermentation on the microbial viability, physicochemical properties and bioactivity of juices were investigated. Characterizing changes and sensory properties of fermented beetroot LJ during storage is promising in expanding the literature, and critical to enable the laboratory studies’ guidance for industrial applications.

## 2 Materials and methods

### 2.1 Materials

Recently harvested fresh leaves of broccoli, cauliflower, beetroot, and black carrot were collected from local markets in Istanbul (Türkiye) in 2024–25 season. All leaves were stored at −20 °C until used, and were thawed just before the analyses. All chemicals used were of analytical grade.

### 2.2 Determination of proximate composition

Moisture, ash, protein and lipid analyzes to determine the proximate composition of the leaves and leaf juices were carried out according to the AOAC standard method (42). Protein content was determined by calculating the amount of crude protein from nitrogen content (P% = N% × 6.25) using the Kjeldahl method.

### 2.3 Production of leaf juices

Leaves were cold pressed by using a twin-screw extruder (GSE-5000-F, Tribest, United States) in order to fractionate the leaves into juice and pulp. LJ yield was calculated by the following equation:


$$\mathrm{LJ}\;\text{Yield}\;(\%)=\frac{\text{Volume of juice}\;(\mathrm{mL})}{\text{Mass of leaves}\;(g)}\times 100$$

The amount of total soluble solids (°Brix) of each LJ was determined using a hand refractometer.

### 2.4 Characterization of LJs

#### 2.4.1 Total phenolic and flavonoid contents of LJs

The total phenolic content (TPC) of LJs was evaluated using Folin–Ciocalteu method (43). 100 μL of sample was mixed with 0.75 mL of 0.2 N Folin–Ciocalteu reagent and kept in the dark for 5 min. 750 μL of saturated Na_2_CO_3_ (6%) solution was added to the mixture. After 90 min, the absorbance of samples was measured at 765 nm by a microplate reader (Epoch 2, BioTek Instruments, United States). The calibration curve was constructed using gallic acid. Results were expressed as mg gallic acid equivalents (GAE)/mL LJ.

For the total flavonoid content (TFC), 1.25 mL of distilled water and 75 μL of 5% (w/v) NaNO_2_ were added on 0.25 mL LJ sample, and incubated for 6 min. Afterwards, 150 μL of 10% (w/v) AlCl_3_·6H_2_O was added, and incubated for 5 more min. After adding 0.5 mL of 1 M NaOH, the volume was completed to 2.5 mL with distilled water. The absorbance of samples was measured at 510 nm by a microplate reader (44). The calibration curve was constructed using quercetin. Results were expressed as mg quercetin equivalents (QE)/mL LJ.

#### 2.4.2 Antioxidant capacity of LJs

The total antioxidant capacity of LJ samples was determined by using both 2,2-diphenyl-1-picrylhydrazyl (DPPH) scavenging activity (45) and cupric reducing antioxidant capacity (CUPRAC) assays (46). For the DPPH assay, 100 μL sample was mixed with 2 mL of a 0.1 mM DPPH solution and kept in dark for 30 min at room temperature. The absorbance of samples was measured at 517 nm by a microplate reader. For the CUPRAC assay, 100 μL sample was mixed with 1 mL of 10 mM CuCl_2_, 1 mL of 7.5 mM neocuprine, 1 mL of NH_4_Ac, and 1 mL of distilled water. After 60 min, the absorbance of the samples was measured at 450 nm by a microplate reader. The calibration curve was constructed using Trolox. Results were expressed as mg Trolox equivalents/mL LJ.

#### 2.4.3 *In vitro* bioaccessibility of LJs

The *in vitro* digestion protocol based on the simulation of sequential gastric and intestinal digestion was conducted as described Minekus et al. (47), with slight modifications. 5 mL sample was mixed with 4 mL of salivary fluid, 25 μL of 0.3 mol/L CaCl_2,_ and 975 μL of distilled water, and the pH was adjusted to 7. The mixture was shaken for 2 min at 37 °C. After that, 7.5 mL of gastric fluid, 1.6 mL of pepsin solution (25,000 U/mL), and 5 μL of 0.3 mol/L CaCl_2_ were added to the mixture, and the pH was adjusted to 3. The mixture was shaken for 2 h at 37 °C. For the analyses of gastric phase, aliquots of 5 mL were collected for each sample before the intestinal digestion. Lastly, 8.25 mL of intestinal fluid, 3.75 mL of pancreatin (800 U/mL), 1.875 mL of bile (0.16 mol/L), and 30 μL of 0.3 mol/L CaCl_2_ were added on the remaining mixture, and the pH was adjusted to 7. The mixture was shaken for 2 h at 37 °C. For the analyses of intestinal phase, aliquots of 5 mL were collected for each sample. All samples from gastric and intestinal phases were centrifuged at 10,000 rpm for 30 min and supernatant were collected. The *in vitro* bioaccessibility was calculated using the following equation:


$$\begin{array}{l} \text{Bioaccessibility}\;(\%)=\\ \frac{\text{Bioactive compounds in the intestinal phase}\;}{\text{Bioactive compounds in the undigested juice}}\times 100 \end{array}$$

#### 2.4.4 Cytotoxicity of LJs

For cell viability, CCK-8 assay (48) was applied. Caco-2 cells were cultured at 37 °C and 5% CO_2_ in DMEM including 4.5 g/L glucose and stable glutamine supplemented with 20% FBS, 1% NEAAs, 1% penicillin/streptomycin. Caco-2 cells were seeded in 96-well plates at a concentration of 10,000 cells in 50 μL of growing medium per well. After 24 h, cells were treated with LJ samples at different concentrations ranging from 0.0625 to 1% (v/v) in growing medium. After 24 h of exposure, 10 μL of CCK-8 reagent was added to each well. After standing 3 h in the dark conditions, the optical density was recorded at 450 nm using a microplate reader. Results were stated as percentage (%) of cell viability relative to control (cells grown in culture medium).

### 2.5 Water kefir fermentation

Water kefir fermentation of LJs was conducted according to Dikmetas et al. (49) Water kefir grains were provided by Danem Inc. (Isparta, Turkey), and kept in a 5% sterile brown sugar solution in a shaking incubator at 25 °C. The brown sugar solution was freshened every 24 h for the maintenance of the viability. Green leaves were washed with tap water, cold-pressed into LJ, and centrifuged at 9000 rpm for 5 min. The supernatants were pasteurized for 5 min at 75 °C. After cooling the pasteurized LJs, kefir grains (%1, w/w) were inoculated. Fermentation was performed at 37 °C for 48 h, with pH monitored at regular intervals. For storage analyses, fermented juice was cold stored at 4 °C for 21 days.

### 2.6 Microbial viability of fermented LJs

The viability of LAB and yeasts in fermented LJs were determined. To determine total yeast count, the spread plate method was used on Yeast Peptone Dextrose Agar (YEPDA), and petri dishes were incubated at 25 °C for 48 h. M17 and Man-Rogosa-Sharpe (MRS) agar were used for coccus and rod-shaped LAB counts. Petri dishes inoculated using the pour plate method were incubated anaerobically at 37 °C for 48 h. Yeast and LAB viability before fermentation, after 48 h of fermentation (storage day 0), and on predetermined days during 21 days of storage.

### 2.7 Sulforaphane content

Sulforaphane was extracted by the methods of Totušek et al. (50) and Martínez-Hernández et al. (51), with slight modifications. 25 mL of LJ was incubated at 45 °C for 2 h and centrifuged at 10,000 rpm for 5 min. The clarified juice was collected and mixed with 10 mL dichloromethane (DCM). The mixture was centrifuged (10,000 rpm, 5 min) and DCM fraction was removed. The remaining part of LJ was mixed with 5 mL of DCM, centrifuged and DCM fraction was separated. This procedure was repeated twice, and all collected DCM fractions were combined. The DCM fraction was filtered through glass wool, and aqueous phase was removed with anhydrous Na_2_SO_4_. Extract was dried by rotary evaporator at 38 °C, and eluted with acetonitrile (52). The eluent was filtered with a 0.45 μm polytetrafluoroethylene syringe filter, and stored in a freezer.

The sulforaphane content was determined by the modified method of Wu et al. (52) with HPLC system (Agilent Technologies 1,100) equipped with a C18 column (250 mm × 4.6 mm, 5 μm, Supelco, United States) at 30 °C. HPLC-grade water (A) and 100% acetonitrile (B) were used at the flow rate of 1 mL/min. The detection was done with a diode array detector (Agilent G1315D) at 240 nm. The standard curve was prepared using sulforaphane standard (S4441, Sigma-Aldrich, United States) stock solution prepared in acetonitrile (53). Analyses were performed before fermentation, after 48 h of fermentation (storage day 0), and on predetermined days during 21 days of storage.

### 2.8 Physicochemical changes

The pH of the samples was determined using a digital pH meter. The titratable acidity (TTA) of the samples was determined using a titrimetric method and expressed using the oxalic acid conversion coefficient. Total soluble solids (TSS, °Brix) were measured using a hand refractometer. The color parameters (L*, a*, b*) of LJs were determined using a colorimeter (Minolta Chroma Meter CR-400, Minolta Co Ltd., Japan). The color change (ΔE) in LJs caused by fermentation was calculated as follows:


$$\mathrm{\Delta E}={\left({\left(L*-L_{0}*\right)}^{2}+{\left(a*-a_{0}*\right)}^{2}+{\left(b*-b_{0}*\right)}^{2}\right)}^{0.5}$$

All analyses were performed before fermentation, after 48 h of fermentation (storage day 0), and on predetermined days during 21 days of storage.

### 2.9 Gamma-aminobutyric acid (GABA) and organic acids content

The concentration of GABA and organic acid profile of beetroot LJ were determined by the method described by Devecioglu et al. (54). A HPLC (Agilent Technologies 1,100) system equipped with a C18 column (250 mm × 4.6 mm, 5 μm, Supelco, United States) and a diode array detector (DAD, Agilent G1315D). The mobile phase (A) was HPLC-grade water containing 0.1% phosphoric acid and mobile phase (B) was 100% methanol. Isocratic flow (97:3, v/v) rate was set at 0.70 mL/min. The detection was done at 210 nm. The standard curve was prepared with GABA, lactic acid, acetic acid, malic acid, tartaric acid standards. All analyses were performed before fermentation, after 48 h of fermentation (storage day 0), and on predetermined days during 21 days of storage.

### 2.10 Sensory analysis

To determine the sensory properties of fermented beetroot LJ throughout storage, an untrained panelist group consist of 15 people from different ages and genders evaluated the product. A 9-point hedonic scale was used, 1 for extremely disliked and 9 for extremely liked, for scoring the attributes including color, turbidity, odor, taste, flavor, sparkling effect and overall acceptability. Scores of each attribute were expressed as mean ± standard deviation.

### 2.11 Statistical analyses

All experiments were analyzed statistically using Minitab (Version 18.1, United States). Each experiment was conducted as three independent replicates and expressed as mean ± standard deviation. The differences between groups were compared by one-way analysis of variance (ANOVA), followed by Tukey’s multiple range test. Differences were considered significant if *p* < 0.05.

## 3 Results and discussion

### 3.1 Characterization of LJs

#### 3.1.1 Proximate composition of leaves and their juices

Prior to green juice extraction and bioactivity analyses, proximate composition of each leaf was determined and the results are given on a dry basis in Supplementary Table S1. The literature on the proximate composition of fruit and vegetable leaves left as waste in the field during harvesting is quite limited. The composition of vegetables may vary depending even on the content of the soil and water source in which they grow (55). Even leaves obtained from the same markets at different times are likely to differ compositionally.

The juice yields obtained by cold press, and their proximate composition are summarized in Table 1. The juice yield of beetroot leaves was significantly higher than that of others (*p* < 0.05), probably due to its lower amount of carbohydrates among other leaves, since the pulp remaining after juice extraction of vegetables mainly consists of dietary fibers (56). The leaf with the highest juice yield, beetroot, also has the lowest total soluble solids content. Considering the volume of leaves produced as waste each year, the fact that at least half of their weight can be utilized as juice through this simple cold-pressing is industrially applicable, practical, and also promising.

**Table 1:** Proximate composition of LJs.

Leaf	Green juice yield (mL/100 g leaf)	°Brix	Protein (%)	Ash (%)
Beetroot	67.20 ± 1.91^A^	6.00 ± 0.00^B^	1.01 ± 0.03^C^	3.80 ± 0.34^A^
Black carrot	51.40 ± 3.03^B^	12.17 ± 0.76^A^	1.41 ± 0.13^BC^	2.85 ± 0.61^B^
Broccoli	54.33 ± 0.58^B^	10.83 ± 0.76^A^	2.58 ± 0.32^A^	2.00 ± 0.11^B^
Cauliflower	52.33 ± 2.52^B^	6.33 ± 0.76^B^	1.56 ± 0.15^B^	0.53 ± 0.22^C^

Values with different letters in the same column are statistically different (*p* < 0.05).

#### 3.1.2 Total phenolic and flavonoid contents of LJs

The bioactivity of LJs in terms of TPC, TFC, DPPH and CUPRAC was calculated per gram of leaf on a dry basis in order to increase the comparability with the literature, and given in Table 2. Beetroot LJ showed significantly higher bioactivity compared to the other leaves (*p* < 0.05), which is not surprising since it stands out with its bioactive component content and preservative capacity among leafy vegetables (57).

**Table 2:** Total phenolic and flavonoid contents and antioxidant capacity of LJs obtained by cold pressing of leaves.

Leaf	TPC (mg GAE/g leaf, DW)	TFC (mg QE/g leaf, DW)	DPPH (mg Trolox/g leaf, DW)	CUPRAC (mg Trolox/g leaf, DW)
Beetroot	7.95 ± 0.69^A^	12.62 ± 0.50^A^	5.01 ± 0.17^A^	28.23 ± 2.38^A^
Black carrot	3.27 ± 0.25^C^	4.09 ± 0.19^B^	1.53 ± 0.03^B^	10.06 ± 0.51^C^
Broccoli	5.38 ± 0.50^B^	3.09 ± 0.28^C^	1.86 ± 0.13^B^	16.00 ± 1.17^B^
Cauliflower	3.41 ± 0.11^C^	2.35 ± 0.23^C^	1.37 ± 0.11^B^	11.02 ± 0.64^C^
Bioaccessibility (%)				
Beetroot	32.63 ± 2.64^a^	26.22 ± 2.49^ab^	44.64 ± 1.19^c^	43.99 ± 3.81^a^
Black carrot	26.95 ± 1.69^a^	23.58 ± 1.38^ab^	53.39 ± 2.73^c^	33.02 ± 1.50^b^
Broccoli	28.79 ± 2.39^a^	30.95 ± 0.88^a^	80.41 ± 4.21^b^	31.48 ± 1.93^b^
Cauliflower	33.30 ± 6.39^a^	21.34 ± 4.92^c^	98.49 ± 7.09^a^	38.17 ± 4.54^ab^

Difference between values with different uppercase letters within the same column are statistically significant (*p* < 0.05). Difference between values with different lowercase letters within the same column are statistically significant (*p* < 0.05).

Previous work was performed using several pretreatments and/or extraction approaches to obtain bioactive components from leaves, usually followed by drying. However, cold pressing was adapted as a sustainable and “green” approach to valorize leaves to explore the potential of use in liquid products such as sauces and beverages. Nonetheless, present work led to higher (11, 23, 25, 58), similar (14), or less (12, 26, 59) concentrations of phenolic compounds, when compared to common solvent extraction obtained from broccoli and cauliflower leaves. Previous studies reported significantly higher TPC values when beetroot leaves were extracted by aqueous ethanol. Separation of stems from the leaves (17, 58), or pre-drying of the leaves before extraction (18, 60) together with the reported effect of region (59), harvesting season (23), plant maturity (11, 22) and even the age and position of the leaf on the plant (61) on the bioactive component accumulation in leaves, are thought to be the reasons for such difference. Also, freezing the leaves after harvest, in order to prevent spoilage during the study period, may have led to a reduction in bioactivity compared to freshly harvested leaves. Black carrot leaves have not been considered as a bioactive components source, as opposed to orange carrot (20–22, 58), until the present study.

The TFC of broccoli LJ was less than the extract obtained by using 70% methanol, 70% ethanol, or hot water (12). Extracts obtained from the sprout and seed were reported to have higher TFC than the leaves in the current study (62). The cauliflower LJ showed both significantly lower (59) and higher (63) TFC values from previous extraction studies, emphasizing the importance of region and the aerial part of the plant. Beetroot LJ in the current study was 2.5- to 12-fold (60) and 10-fold (18) richer in TFC than the extracts obtained from beetroot leaves with different solvents and conditions, and up to 10-fold higher than organic and conventional beetroot juices (64). TFC of black carrot LJ, unsurprisingly, was significantly higher than ethanol and hot water extracts from carrot leaves (65), probably due to the higher flavonoid concentration of black carrot compared to orange or red carrots (66). For future research, the phenolic and flavonoid content of the pulp remaining after LJ production could also be examined, as polyphenols can transfer into by-products as pulp or flour in high amounts, up to 20% (67).

#### 3.1.3 Antioxidant capacity of LJs

The notably higher CUPRAC values compared to DPPH values are related to the difference in these assays’ mechanisms. CUPRAC assay detects both lipophilic and hydrophilic antioxidants, while DPPH assay detects only lipophilic ones; moreover, CUPRAC has a structure that is less affected by external factors (68). According to the results in Table 2, the fact that CUPRAC values are statistically higher than DPPH suggests that antioxidant activity in LJs is mainly provided by hydrophilic compounds (*p* < 0.05). In both approaches, the juice with the highest antioxidant activity was obtained from beetroot leaves, followed by broccoli leaves. The antioxidant activity of broccoli juice was found to be lower than both broccoli stems’ and leaves’ extracts (26). It has been reported that the antioxidant activities of broccoli, cauliflower, beetroot and their wastes vary depending on the part of the plant (12, 63), the genotype (69, 70) together with the region the plant grew (59), the extraction method, conditions and the solvent used (14, 24, 29). It was expected that the antioxidant activities of LJ produced from black carrot leaves would be lower than those of black carrots (71); they even have a higher capacity than purple and orange carrots (72) and this can be associated with their color.

Although phenolics and flavonoids are the compounds that primarily contribute to the antioxidant activity of plants, the contribution of compounds such as anthocyanins and carotenoids is undeniable (73), and antioxidant activity is evaluated to better examine the combined effect of all bioactive compounds (74). TPC and TFC are both relevant, but TPC has a higher positive correlation with DPPH antioxidant activity (73), explaining the same order of elevation of TPC and DPPH values in selected leaves in the current study. It can be interpreted as the dominance of the water-soluble substances among flavonoids in the leaves, where the antioxidant activity determined by CUPRAC was higher than that of DPPH, and the TFC content increased in the same order as CUPRAC.

#### 3.1.4 *In vitro* bioaccessibility (%) of LJs

The bioaccessibility of bioactive compounds are related to leaf composition or flavonoid content. The food matrix has a significant contribution to its biological activity, where proteins, dietary fibers and minerals are known to be disadvantageous against bioavailability of flavonoids, presumably causing entrapment (75). The bioaccessibility (%) of the LJs is given in Table 2. The undigested samples always had significantly higher polyphenols and flavonoids content when compared to stomach and intestine fluids, which can be explained by the metabolization and transformation of polyphenols to various compounds and interaction with the other food components during digestion (76) by the effect of digestive enzymes, temperature and pH variations (77).

Broccoli LJ had lower bioaccessible polyphenols after *in vitro* digestion, yet, more antioxidant bioaccessibility than a raw broccoli extract (76) and broccoli hydroponic microgreens (78). While the higher polyphenol bioaccessibility of cauliflower LJ than broccoli LJ was consistent with García-Pérez et al. (79), the flavonoid bioaccessibility had the opposite trend. The TPC, TFC and CUPRAC bioaccessibility of beetroot LJ were found to be comparable; however, the recovery of antioxidant capacity determined by DPPH assay was nearly half of the red beetroot juice (80). The investigated four bioactivity indicators’ bioaccessibility for beetroot LJ were drastically higher than both fresh red beetroot and its juice (81). The black carrot LJ had considerably higher bioaccessibility in terms of DPPH than black carrot varieties itself; however, the reduction of phenolic content after *in vitro* digestion was higher (82). When Pereira-Caro et al. (71) investigated the polyphenol profile of black carrot, even though there were components whose amount increased or decreased after *in vitro* gastric and intestinal digestion, they stated that the total polyphenol bioaccessibility was 113%, which was higher than processed black carrot products. Considering that black carrot leaves were used in the current study, it is not surprising that the bioactivity of vegetable containing intense pigment was found to be higher than the leaf. In the same study, the DPPH antioxidant bioaccessibility of black carrot was significantly lower than black carrot LJ, while only 0.5% of anthocyanins remained after digestion.

When all bioavailability results were examined, the disruptive effect of *in vitro* digestion was higher in TPC and TFC compared to the antioxidant capacity of LJs, especially in the DPPH assay. The underlying reason for this result may be that phenolic compounds undergo isomerization or degradation during *in vitro* digestion, while antioxidant compounds transformed into new products with similar or higher antioxidative capacity than their precursors (68). Also, the reason why antioxidant recovery was detected higher with DPPH may be that lipophilic antioxidant substances in LJs were more durable than hydrophilic ones during *in vitro* digestion, considering that DPPH detects only lipophilic compounds, unlike CUPRAC.

#### 3.1.5 Cytotoxicity of LJs

**Figure 1:**
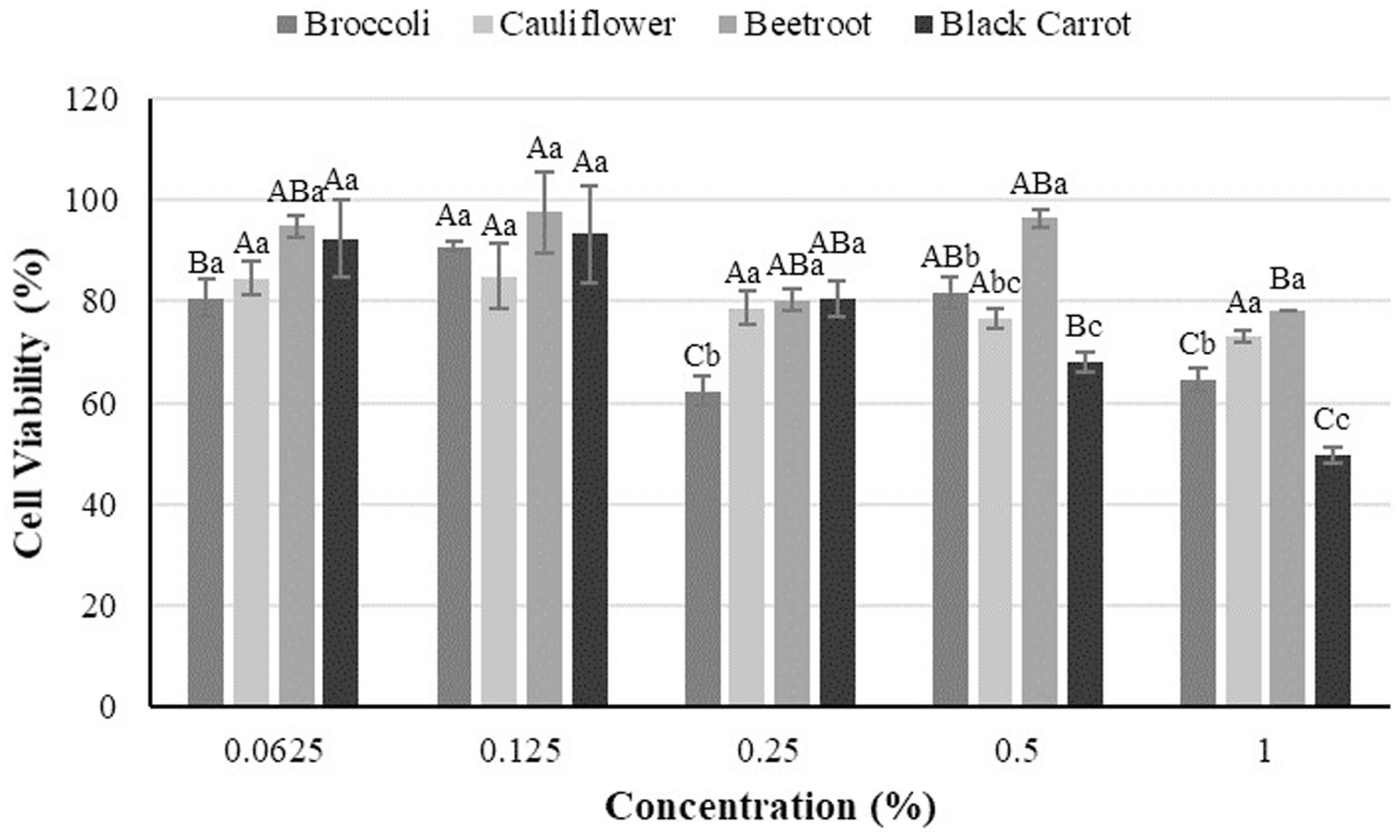
Cytotoxicity of LJs obtained by cold pressing of leaves. Values with different uppercase letters in the same LJ are statistically different (*p* < 0.05). Values with different lowercase letters in the same concentration are statistically different (*p* < 0.05).

The effects of LJs on the Caco-2 cell viability are given in Figure 1. All results varied between 50–98%, some with less effect on viability. While there was no statistically significant difference between leaves at the lowest concentration, cytotoxicity differed depending on the leaves as the concentration increased. The least cell death at almost all concentrations belonged to beetroot LJ.

To the best of our knowledge, the current study is the only one examining the cytotoxicity of green leaves as agricultural waste. Although the literature contains many studies examining the effects of medicinal plants and their leaves on cell viability, evaluating the juice of waste green leaves is a new perspective, which enables the direct use of LJs without additional extraction. Previous study reported that the floret, leaf and seed extracts of broccoli did not affect the normal liver cells (FL83B) while showing stronger cytotoxic efficacy toward lung carcinoma (A549), colorectal adenocarcinoma (Caco-2) and hepatocellular carcinoma cells (HepG2) (12). Methanolic extracts of broccoli leaves showed anticancer activities against NCI-H1299 cell lines, varying with harvest time (11). Although the order may vary at different concentrations, cauliflower LJ caused less cell death than that of broccoli. As opposite, Kassie et al. (83) found crude broccoli juice less genotoxic than cauliflower juice against *Escherichia coli* K-12 cells. For the cosmetic and pharmaceutical products, the cytotoxicity of extracts from various berry leaves was examined both for keratinocytes and fibroblasts, indicating the dependence of cytotoxicity with the concentration used (84), which supports our results. Cytotoxicity results were parallel to the total soluble solid content of LJs since the lowest cytotoxicity was detected in the LJ with the lowest °Brix value or vice versa. This indicates that toxicity may be related to the soluble solids content. For a better understanding of the relationship between these two properties, effects on cell metabolism should also be examined. In order to expand the literature, the cytotoxicity of LJs on different cell lines can be examined, but it is recommended to support them with allergenicity tests before they are offered for human use.

### 3.2 Leaf selection for fermentation by water kefir grains

Based on the analyses performed, beetroot LJ fermented using water kefir, which showed the highest LAB and yeast viability, and sulforaphane content, while having the least cytotoxicity, was selected for further examination, including cold storage and sensory analysis. Leaf selection is discussed in detail below.

#### 3.2.1 Microbial viability of fermented LJs

In this study, it was planned to select one of the four LJs analyzed for fermentation with water kefir grains, and investigate it during cold storage. When all LJs were pasteurized, large precipitates were observed in broccoli LJ, perhaps due to its higher protein content compared to others (*p* < 0.05), and water kefir was not inoculated into broccoli LJ as this could subsequently reduce consumer acceptance. When beetroot, black carrot, and cauliflower LJs were fermented with water kefir for 48 h, the highest viability of all *Lactobacillus* spp. *Lactococcus* spp. and yeasts were observed in beetroot LJ (*p* < 0.05).

The microbiota of water kefir generally consists of LAB and yeasts. In the grains, LAB may be dominant over yeast, present in equal or smaller amounts, and this diversity is linked to their origins in different geographic regions (37). Based on the microbial profile at 0 h of fermentation, i.e., the moment of inoculation, the grains used in this study have a yeast-dominant microflora. During fermentation, the microbial populations in each LJ became balanced, and *Lactobacillus* spp., *Lactococcus* spp., and yeast counts reached nearly equal levels despite differences in total numbers between the LJs. In fermented LJs, coccus and rod-shaped LAB showed similar growing trends in all juices, similar to many studies in the literature (41, 49, 85). However, although LAB growth was also less than others, the yeast growth trend in cauliflower LJ was significantly the least compared to others (*p* < 0.05). This is consistent with a study that fermented different vegetable juices and found the lowest yeast viability in an onion kefir-like beverage, which was explained by the fact that high amounts of sulfur compounds inhibit different yeast species, such as *Saccharomyces cerevisiae* (85). Furthermore, yeast growth in the media directly affects the growth of LAB, and the relatively low LAB counts in cauliflower LJ may be attributed to this. Yeasts, especially *Saccharomyces* species, hydrolyze sucrose found in fruit and vegetable juices into glucose and fructose by their invertase enzyme, thus releasing carbon sources in the media that LAB can utilize (86). Despite the differences, a food must contain at least 10^6^ CFU/g probiotic microorganisms to be considered probiotic (87), and 48-h of fermentation resulted in enough microbial growth in all three LJ samples to meet this requirement.

#### 3.2.2 Sulforaphane content

Sulforaphane, a hydrolysis product of glucoraphanin, has gained importance due to its physiological effects, such as being antidepressant, anti-inflammatory and hypoglycemic, and the preventative effect against various types of cancer and intestinal diseases (88). It also protects central nervous system by crossing the blood–brain barrier; however, low stability and incomprehensibility of the molecule have limited the widespread use (89). Considering the health advantages arising from its consumption, literature on sulforaphane content is considered to be limited and studies should be intensified.

**Figure 2:**
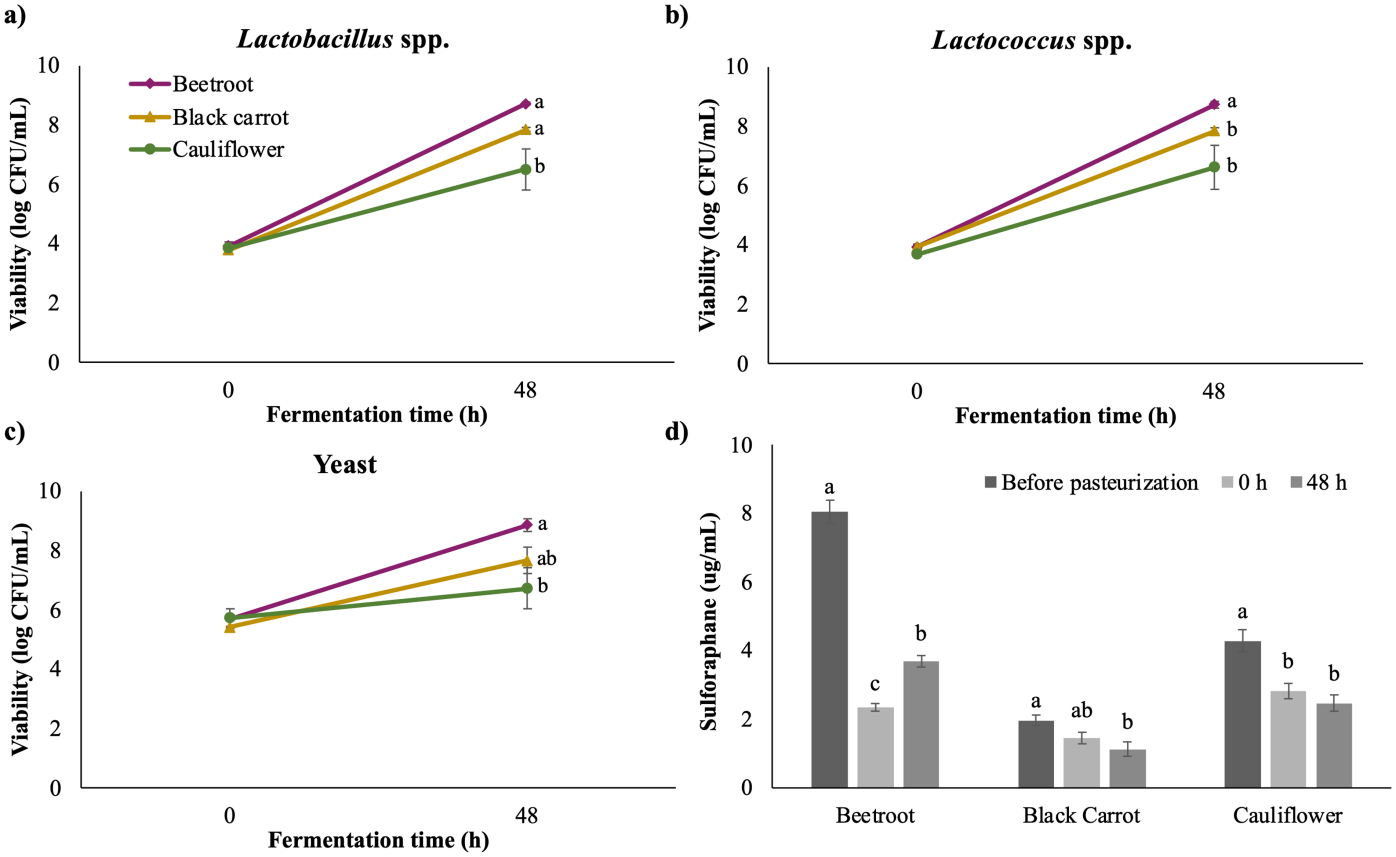
**(a)** *Lactobacillus* spp.; **(b)** *Lactococcus* spp.; **(c)** Yeast viability (log CFU/mL) of LJs at 0 h and 48 h of water kefir fermentation; **(d)** Sulforaphane content (μg/mL) of LJs. The times indicated as 0 h and 48 h refer to the 0th and 48th hours of fermentation. Differences between values with different letters within graphs a–c are statistically significant (*p* < 0.05). Different letters within the same LJ in graph d represent statistically significant difference (*p* < 0.05).

The sulforaphane content of LJs was determined right after; (i) cold-pressing (before pasteurization), (ii) pasteurization and inoculation with grains (0 h), and (iii) 48 h of water kefir fermentation (Figure 2). The sulforaphane concentration of fresh broccoli LJ (11.42 ± 0.63 μg/mL) was the highest among all (*p* < 0.05). However, due to the visual changes occurred in pasteurization, other three LJs were subjected to fermentation, and the highest concentration was recorded in the fresh beetroot LJ (*p* < 0.05). Recent studies showed that while the fresh broccoli stalks fractions contained 10-fold higher sulforaphane than the LJ, the lyophilized or dried samples showed more than 10-fold reduced sulforaphane content (88). The amount of sulforaphane in broccoli LJ was lower than the juice of fresh broccoli sprouts (90), comparable with lyophilized broccoli byproducts (91), and higher than broccoli juice obtained under various treatment conditions (92). These variations in the amount of sulforaphane are expected since the content is known to vary depending on harvest location, variety (11), part of the plant used, whether the leaf is fresh or frozen (91), applied treatments including temperature and pressure (50), and even whether the obtained juice is clear or cloudy (90). The higher sulforaphane detection in broccoli LJ compared to cauliflower LJ is consistent with the literature, but the sulforaphane concentration of LJ in the present study was higher than the juice treated with various temperature applications (50). Broccoli, Brussels sprouts, cauliflower, and cabbage, as members of cruciferous vegetables, are known to be among the few vegetables that contain sulforaphane (91). Surprisingly, beetroot LJ had a sulforaphane content between broccoli and cauliflower LJs, while being statistically similar to broccoli in terms of leaf weight on a dry basis. To the best of our knowledge, the sulforaphane content of beetroot and black carrot leaves and/or juice is lacking in the literature, but its undeniable amount, especially in beetroot LJ, should be evaluated in future studies.

Pasteurization significantly reduced the sulforaphane concentration in LJs (*p* < 0.05). Sulforaphane is an unstable compound at high temperatures (93), and undergoes decomposition when exposed to thermal processing in aqueous solutions at temperatures of 50–100 °C (94). However, fermentation also had a significant effect on the concentration of sulforaphane in LJs (*p* < 0.05). There are studies in the literature that report both a reduction (95) and an increment in the sulforaphane content after fermentation (96). Although sulforaphane decreased in black carrot and cauliflower LJs after fermentation, it increased in beetroot LJ, which had the highest sulforaphane concentration in fresh leaf. Since LAB can increase the hydrolysis of the glucosinolates, the sulforaphane concentration may increase during fermentation (97). Furthermore, sulforaphane is more stable under acidic conditions (93). The fact that the pH of beetroot LJ after fermentation was lower than that of cauliflower may be one reason for this increase in beetroot LJ.

#### 3.2.3 Total phenolic and flavonoid contents and antioxidant capacity of fermented LJs

The results of TPC, TFC, CUPRAC and DPPH assays of water kefir fermented LJs are given in Table 3. While fermentation tended to increase the bioactivity of beetroot and black carrot LJs, TPC, TFC and the antioxidant capacity of cauliflower LJ were significantly decreased after fermentation (*p* < 0.05).

**Table 3:** Total phenolic and flavonoid contents and antioxidant capacity of water kefir fermented LJs.

Fermentation time	TPC (mg GAE/mL)	TFC (mg QE/mL)	DPPH (mg Trolox/mL)	CUPRAC (mg Trolox/mL)
*Beetroot*				
0 h	8.88 ± 0.14^A^	2.05 ± 0.07^A^	4.53 ± 0.16^A^	2.68 ± 0.02^A^
48 h	8.97 ± 0.07^A^	2.15 ± 0.05^A^	4.71 ± 0.13^A^	2.78 ± 0.13^A^
*Black Carrot*				
0 h	10.71 ± 0.15^A^	2.66 ± 0.08^B^	5.41 ± 0.06^A^	4.21 ± 0.05^A^
48 h	11.00 ± 0.19^A^	3.22 ± 0.11^A^	4.63 ± 0.07^B^	4.29 ± 0.09^A^
*Cauliflower*				
0 h	6.46 ± 0.07^A^	0.56 ± 0.04^A^	2.61 ± 0.06^A^	1.19 ± 0.04^A^
48 h	5.62 ± 0.14^B^	0.39 ± 0.08^B^	1.73 ± 0.11^B^	1.12 ± 0.10^A^

The times indicated as 0 h and 48 h refer to the 0th and 48th hours of fermentation. Difference between values with different uppercase letters within the same column for the same leaf are statistically significant (*p* < 0.05).

Phenolic and antioxidant compounds can undergo different pathways for various reasons; therefore, the literature includes examples of both increased and decreased bioactivity during fermentation. As in beetroot and black carrot LJs, studies report an increase in TPC, TFC and/or antioxidant compounds after fermentation are frequent (35, 49, 98), since the fermentation promotes the hydrolysis of glycosidic bonds in phenolic compounds, leading to the release of bound phenolics and their conversion into additional, often more bioactive phenolic metabolites (99, 100). On the other hand, a study investigating both the pomace and juice of aronia reported a reduced TPC, TFC and antioxidant activity (38). They explained the reduction by the consumption of phenolic and antioxidant compounds by LAB as a nutrient for their growth, the modifications made by the microbial enzymes, and the pH-dependent instability of bioactive compounds during fermentation.

#### 3.2.4 Physicochemical changes at LJs after fermentation

The physicochemical properties of LJs before and after fermentation are given in Table 4. In all samples, the pH decreased while the TTA increased after fermentation. These changes were significant in both beetroot and black carrot LJs (*p* < 0.05). During fermentation, microorganisms increase the acidity of the environment and lower its pH by producing and releasing various organic and fatty acids, particularly lactic acid (101, 102). In many foods, lowering the pH is critical in order to reduce the risk of pathogens. Specifically, since botulinum toxin production is not observed at pH values below 4.6 (103), storing foods below this value is considered safe. After fermentation, all LJs except cauliflower reached the safe pH range, which was taken into consideration when selecting the leaf type for the plant-based fermented beverage.

**Table 4:** Physicochemical properties of water kefir fermented LJs.

Fermentation time	Beetroot	Black carrot	Cauliflower
*pH*			
0 h	7.65 ± 0.01^Aa^	5.75 ± 0.01^Ba^	5.51 ± 0.01^Ca^
48 h	4.48 ± 0.11^Bb^	4.32 ± 0.12^Bb^	5.22 ± 0.36^Aa^
*TTA (g oxalic acid/L)*			
0 h	0.29 ± 0.01^Cb^	1.21 ± 0.03^Ab^	1.02 ± 0.05^Ba^
48 h	1.27 ± 0.09^Ba^	2.17 ± 0.06^Aa^	1.06 ± 0.09^Ba^
*TSS (°Brix)*			
0 h	5.30 ± 0.26^Ba^	6.47 ± 0.06^Aa^	5.83 ± 0.29^Ba^
48 h	4.97 ± 0.06^Ba^	5.30 ± 0.26^ABb^	5.76 ± 0.25^Aa^
Color			
ΔE	5.51 ± 0.60^A^	4.53 ± 0.32^A^	3.24 ± 0.33^B^

The times indicated as 0 h and 48 h refer to the 0th and 48th hours of fermentation. Difference between values with different uppercase letters within the same row are statistically significant (*p* < 0.05). Difference between values with different lowercase letters within the same column for the same parameter are statistically significant (*p* < 0.05).

The TSS in LJs tended to decrease due to water kefir fermentation. This was expected since LAB ferment sugars into organic acids, as a part of their metabolism (86, 98). The least decrease in °Brix values was recorded in cauliflower LJ, which was in consistence with the results of pH and TTA, indicating the fermentation was slower, probably due to the high sulfur content. The pH, TTA, and TSS trends were parallel with studies in the literature on the water kefir fermentation of various fruit or vegetable juices (35, 49, 85).

The change in color of the LJs (ΔE) was calculated using the samples before and after fermentation. Similar to previous results, the most apparent color change was recorded in beetroot LJ, which was thought to be the fastest fermented one due to its higher LAB and yeast counts, pH and TTA variation. Although the color change was the least in cauliflower LJ (*p* < 0.05), fermentation caused a noticeable color change in all LJs, considering the visible limit is 2.3 (104).

### 3.3 Changes in water kefir fermented beetroot LJ throughout storage

#### 3.3.1 Microbial viability

**Figure 3:**
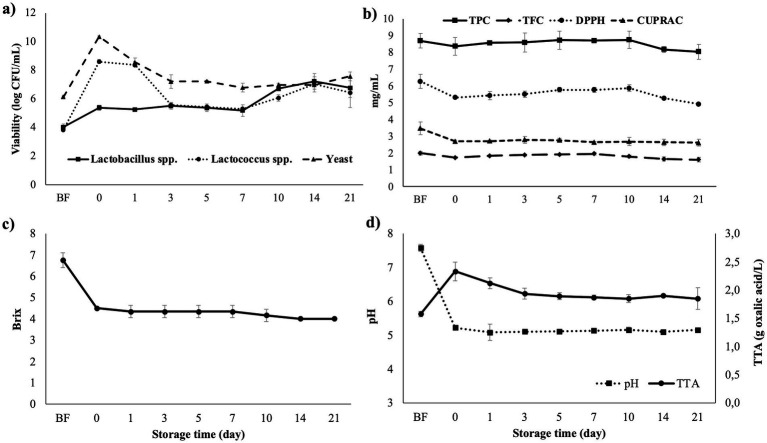
**(a)** The *Lactobacillus* spp., *Lactococcus* spp. and yeast viability (log CFU/mL); **(b)** TPC, TFC and antioxidant activity; **(c)** total soluble solids (°Brix); **(d)** pH and TTA of fermented beetroot LJ during 21 days of cold storage. BF indicates before fermentation.

The beetroot LJ was fermented with water kefir grains for 48 h, and stored at 4 °C for 21 days. The microbial viability curve during storage is given in Figure 3a. Before fermentation, the yeast count was more abundant than LAB, and it maintained the dominance during the first week of storage. The *Lactobacillus* and *Lactococcus* populations increased and the microbiota became more homogenous starting from the 10th day. The maximum *Lactococcus* spp. (8.61 ± 0.14 log CFU/mL) and yeast counts (10.35 ± 0.04 log CFU/mL) were detected right after pasteurization, on the 0^th^ day of storage. However, *Lactobacillus* spp. reached its highest viability (6.77 ± 0.67 log CFU/mL) at the end of the storage period.

The number of LAB and yeasts in the fermentation media may not show a regular increase or decrease trend during storage. When Figure 3a was examined, a decrease in their numbers was observed after the first few days of storage, followed by an increase, especially from the 10^th^ day onwards. In line with our findings, Koh et al. (105) reported an increase in LAB and yeast viability in pumpkin based coconut water kefir beverage in prolonged storage time at 4 °C. Similarly, although the *Lactobacillus* spp., *Lactococcus* spp. and yeast contents of almond water kefir decreased on day 7, their concentrations increased again on day 14 of storage (101). Ozcelik et al. (41) also reported a decrease in *Lactobacillus* spp., *Lactococcus* spp. and yeast counts in cornelian cherry and hawthorn water kefir beverages at the beginning of storage, while they reached maximum viability on the 28th day of cold storage. They explained it by the synergistic relationship between yeasts, lactic and acetic acid bacteria, discussing that it may be related to the occurrence of two types of fermentation due to the mixed microbiota of water kefir grains, and the organic substances, especially produced by heterofermentative LAB, promote yeast growth. Since the number of microorganisms increased during a period of storage, it is likely that cold-tolerant psychotropic strains were present in the water kefir microbiota. Furthermore, the increase in LAB count after 1 week may be due to the metabolism of yeast fermentation products by the LAB (86), as discussed earlier. In other words, fermentation slowly continued in the 4 °C environment, primarily driven by yeasts, and the accumulated metabolites may have led to an increase in the number of other fermentative microorganisms as well.

#### 3.3.2 Bioactivity

The bioactivity of fermented beetroot LJ in terms of TPC, TFC and antioxidant activity was determined at predetermined days of cold storage (Figure 3b). The effect of fermentation on phenolic and antioxidant compounds was discussed above in detail. No significant changes in bioactivity were observed during storage from days 0 to 10. A significant decrease in TFC and especially antioxidant activity determined by DPPH assay was recorded after fermentation, and 10th day of storage, when microbial growth increased (*p* < 0.05). Ozcelik et al. (41) reported both significant and not-significant decrease in TPC and antioxidant activity of different fruit juices fermented with water kefir during storage. The decrease in phenolic compounds and antioxidant activity can be related to the metabolic and enzymatic activities of LAB, which degrade those bioactive components (38). However, even when the phenolic concentration is reduced, fermentation is known to increase the bioavailability and bioaccessibility of phenolics present in the food matrix (99). Additionally, rather than spontaneous fermentation, the conversion of bioactive phenolic compounds has begun to improve when recombinant microorganisms are used outside of the food matrix (100).

#### 3.3.3 Physicochemical properties

The TSS and pH values decreased, and TTA increased significantly due to fermentation (*p* < 0.05; Figures 3c,d). Similar to other results, the decrease in pH and TSS, and increase in TTA were accelerated after 10 days of storage, when LAB development increased. This result was expected since the LAB and yeasts metabolize sugars into organic acids during fermentation, which led to an increase in acidity while decreasing the pH (98). The accumulation of organic acids in the beetroot LJ supports this finding (Table 5). On the other hand, during cold storage, the sharp reduction after fermentation slowed down and only slight changes were observed, probably because fermentation occurred slowly during the first week of cold storage due to the decreased metabolic activity at low temperatures (96). These slow reducing or increasing trends were in consistence with the literature (39, 105, 106). In addition to providing information about fermentation and microbial growth, physicochemical analyses are also critical for interpreting the sensory characteristics of the beverage. The physicochemical properties directly affect the consumer preferences since °Brix is positively correlated with sweetness, aroma, and color, while pH and TTA are related to the acidity, alcohol perception and texture (86).

**Table 5:** The sulforaphane (ug/mL), gamma-aminobutyric acid (mg/mL) and organic acid (mg/mL) concentrations in water kefir fermented beetroot LJ during cold storage.

Storage time (day)	Sulforaphane (ug/mL)	GABA	Tartaric acid	Lactic acid	Acetic acid	Citric acid
BF	2.74 ± 0.03^e^	23.12 ± 0.01^a^	8.86 ± 0.00^a^	ND^c^	ND^d^	ND^d^
0	3.83 ± 0.53^de^	16.87 ± 0.25^b^	7.69 ± 0.02^ab^	1.19 ± 0.04^bc^	2.95 ± 0.09^c^	2.43 ± 0.04^b^
1	4.64 ± 0.07^cd^	16.01 ± 1.52^bc^	7.85 ± 0.45^ab^	4.93 ± 0.07^a^	10.74 ± 0.67^a^	3.96 ± 0.43^a^
3	5.45 ± 0.05^c^	16.68 ± 0.38^b^	7.89 ± 0.71^ab^	4.29 ± 0.66^a^	4.71 ± 0.36^b^	1.50 ± 0.60^bc^
5	5.72 ± 0.13^c^	16.06 ± 0.34^bc^	7.26 ± 0.39^b^	1.75 ± 0.37^b^	3.16 ± 0.03^c^	1.25 ± 0.05^c^
7	7.24 ± 0.09^b^	14.98 ± 0.75^cd^	8.53 ± 0.22^a^	2.13 ± 0.07^b^	2.94 ± 0.19^c^	1.04 ± 0.04^cd^
10	7.38 ± 0.21^b^	14.83 ± 0.08^cd^	8.56 ± 0.29^a^	2.16 ± 0.51^b^	3.16 ± 0.10^c^	0.85 ± 0.18^cd^
14	8.61 ± 0.67^a^	14.71 ± 0.47^cd^	8.61 ± 0.18^a^	2.19 ± 0.21^b^	3.47 ± 0.45^c^	0.88 ± 0.11^cd^
21	9.59 ± 0.94^a^	14.14 ± 0.15^d^	8.48 ± 0.23^ab^	2.43 ± 0.08^b^	3.36 ± 0.16^c^	1.25 ± 0.10^c^

Difference between values with different lowercase letters within the same column are statistically significant (*p* < 0.05). ND indicates not detected.

#### 3.3.4 Sulforaphane, GABA and organic acids content

The sulforaphane and organic acid concentrations during 21 days of storage are given in Table 5. After fermentation, concentration of sulforaphane increased significantly (*p* < 0.05). During storage, the sulforaphane concentration showed an increasing trend, and at the end of the storage, the sulforaphane content was even higher than the freshly pressed beetroot LJ. As discussed before, during fermentation, LAB can hydrolyze glucosinolates as part of their metabolism, and sulforaphane can be released to the environment (97). It was thought that fermentation continued slowly, even in cold conditions. Furthermore, the concentration of sulforaphane increased after day 10, when the number of LAB also increased, which supports the hypothesis. A study investigated the sulforaphane content in fermented broccoli puree during 4 °C and 25 °C storage (96). They found that fermentation doubled the sulforaphane content compared to fresh broccoli. Also, they stated that sulforaphane was degraded in the fermented broccoli stored at 25 °C, while its concentration remained unchanged for 2 weeks at 4 °C, indicating that it was balanced through simultaneous degradation and generation. Tian et al. (94) also reported that even the water content significantly affects the sulforaphane stability, storage temperatures of −20 °C to 4 °C are suitable for stable storage. In addition to the continuation of fermentation during cold storage, the higher stability of sulforaphane at low pH and low temperature due to the slower degradation reactions (93, 96) may also have contributed to this enrichment.

GABA is a non-protein amino acid widely present in nature, acting as an inhibitory neurotransmitter in the animal central nervous system and a stress-protective compound in plants, and is increasingly produced via sustainable fermentation processes, which has led to its recognition as a promising postbiotic with reported benefits for mood regulation, neural diseases and sleep (54). Since the presence of glutamic acid in leaf protein concentrates obtained from beetroot LJ is reported in our previous work (107), the amount of GABA was investigated during the fermentation and storage processes of beetroot LJ. Instead of an increment, the GABA concentration significantly decreased due to fermentation (*p* < 0.05). The reason behind this reduction was the utilization of GABA by microorganisms, especially *Saccharomyces cerevisiae*, for the maintenance of their viability (108). The water kefir grains alone failed to produce GABA, consistent with Scarpelin et al. (109). Since the substrates for GABA production are glutamic acid and monosodium glutamate, addition of these compounds in the formulations would provide more suitable fermentation media for GABA production (109). By the use of specific GABA-producing LAB strains, it is possible to obtain increased GABA amounts through fermentation under controlled temperature, time and pH conditions (110). In this study, the use of additional ingredients or processes was avoided because functionality was targeted using the simplest processing methods for industrial practicality.

Organic acids exhibited different behaviors depending on the component. For example, the formation of lactic and acetic acid was detected as a result of fermentation and continued during storage. While there were slight changes in tartaric acid concentration, the initial 0.05 mg/mL malic acid was depleted after 21 days of storage. The formation of both lactic and acetic acid demonstrated that the water kefir microbiota consisted of both homofermentative and heterofermentative species, since they produce only lactic acid or lactic acid together with acetic acid, ethanol, and carbon dioxide, respectively (49). The increase in lactic and acetic acid, particularly on day 10, was consistent with microbial growth and the interpretation that fermentation also occurred during storage. Although the citric acid concentration increased during fermentation, it significantly decreased after the first day of storage. Fermentation may result in a reduction of citric and tartaric acid contents due to the LAB-mediated breakdown into lactic acid, and conversion by tartaric acid dehydratase to lactic and acetic acid, respectively (111). The fluctuation of the concentrations of lactic, citric and malic acid during storage was also reported during the storage of water kefir fermented red pitaya beverage (39). The reason behind the increase in malic acid content until the first day of storage may be the *Saccharomyces cerevisiae* present in the water kefir microbiota, a yeast that has the ability of malic acid production (86). However, since some bacteria can decarboxylate malic acid into lactic acid and carbon dioxide (105), the malic acid concentration decreased at prolonged storage times.

#### 3.3.5 Sensory properties

**Figure 4:**
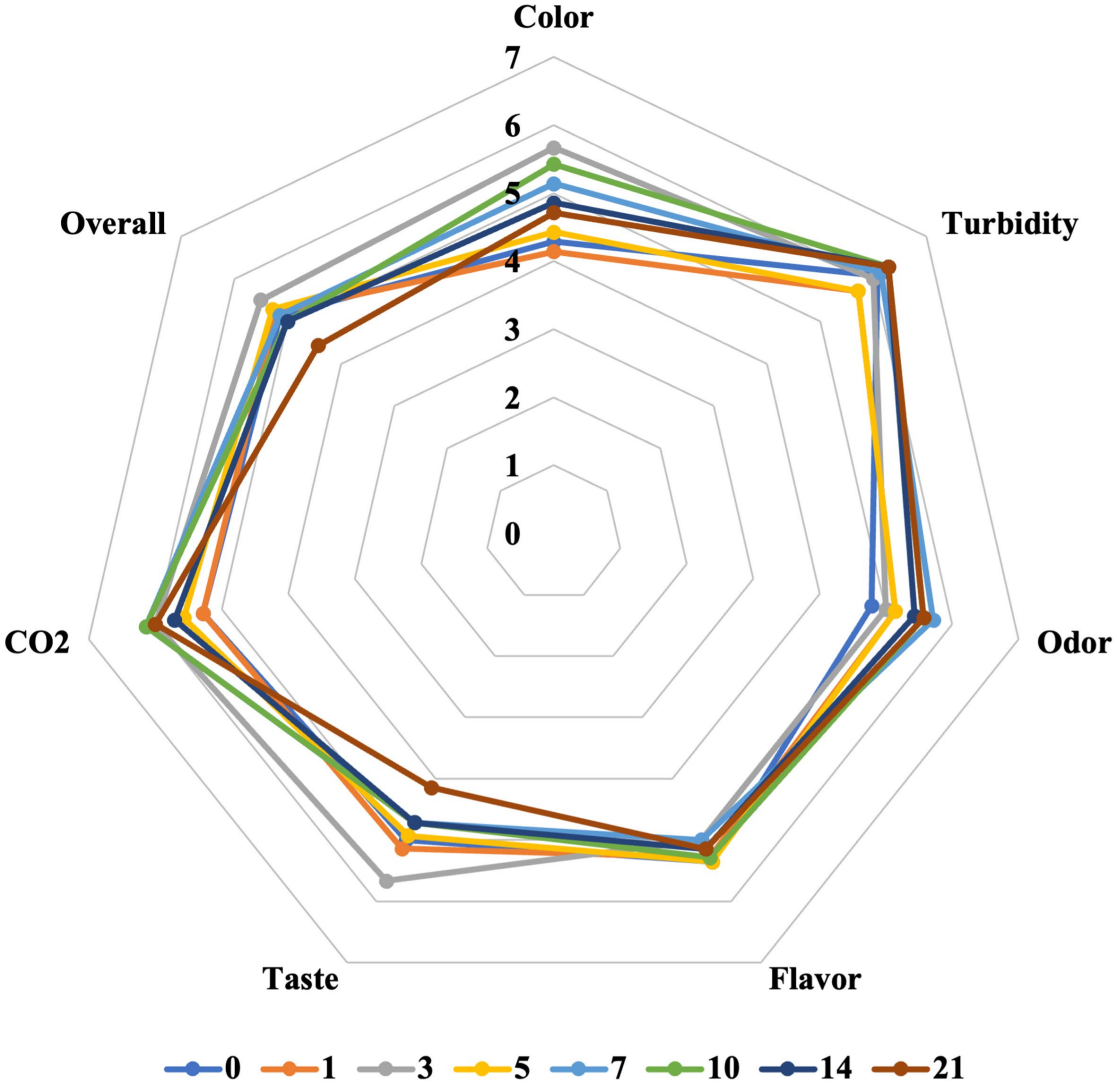
The sensory properties of water kefir fermented beetroot LJ during 0–21 days of cold storage.

The metabolic activity of probiotic bacteria has been reported to have a negative influence on sensory properties, and despite their potential health benefits, consumer acceptance of probiotic foods and beverages largely depends on maintaining desirable taste characteristics (105). It is worth noting that microbiota directly affects the quality, flavor and consumer acceptability of fermented products (112). To assess the consumer acceptability of beetroot LJ fermented with water kefir grains, a sensory analysis was conducted with untrained panelists at the predetermined storage days for the analyses (Figure 4). It is known that products fermented by kefir have a unique flavor due to the ethanol and carbon dioxide produced by yeasts during the fermentation (106). Taken into consideration of the unique compounds released after fermentation as well as having an untrained panel, the evaluation parameters were selected as color, turbidity, odor, flavor, taste, sparkling effect (CO_2_), and overall acceptability.

A hedonic scale of 0–9 was used, and overall, all parameters scored above average on all storage days. While some parameters showed less variation depending on the storage days, color, taste, and overall acceptance scores significantly differed as storage time increased. With these varying parameters, the highest score was recorded on the 3rd day of storage, while at the end of 3 weeks, there was a significant decrease in taste score, and therefore, in overall acceptability. However, the overall acceptability score was below average (4.43) only on the last day of storage. Consumption of this fermented beetroot LJ generally considered preferable for up to 2 weeks after fermentation, but it was still within acceptable limits even in the third week.

The sugar levels reduced at the end of the fermentation, and this caused a reduction in the sweetness scores of fermented aronia juice (38). In the current study, there was no sweetness scale since the LJ was not as sweet as fruit juice. However, as storage time increased, panelists reported a salty taste. This may be because the sugar sources present in the LJ depleted by fermentation, revealing the salty flavor. Also, when lactic, malic, and citric acids are present in juice, sweetness is perceived less strongly (39). Organic acids have a large impact on the organoleptic properties of juices. Our results were consistent with previous findings, which reported that although the literature suggests acetic acid is often associated with unpleasant flavor, this effect was not observed in their study (98). Acetic acid was more abundant than lactic acid in fermented and stored beetroot LJ, however, especially in the first week, both taste and flavor were scored above 5. In a study which different fruit juices were fermented, all water kefir beverages were mildly acidic, mildly alcoholic, and contained gas/CO_2_ as general descriptive criteria (41). Similarly, in our study, sparkling/CO_2_ was quite pronounced, and its score increased as the storage days increased.

This study avoided additional processing steps as it involved upcycling the leaves with minimal processing. However, to prevent a decrease in overall acceptance after 21 days of storage, it is possible to remove any precipitates by appropriate membrane filtration after fermentation, reduce the oxidation of phenolics by adding antioxidants, or achieve controlled aroma development by using a specific LAB strain. Given that fermentation continues slowly at low temperatures, storage at lower temperatures or post-fermentation pasteurization could be considered, but this was not implemented as it would reduce probiotic activity. Nevertheless, this study showed that utilizing green leaf wastes in the production of functional plant-based fermented beverages can be highly preferred by consumers. Overall, the evaluation parameters and acceptability scores were above average, suggesting promise for direct consumption of fermented LJ. However, the uses of LJ could be expanded to include salad dressings, and it is possible to valorize leafy wastes as upcycled beverages or ingredients.

## 4 Conclusion

Although studies on the valorization of food processing wastes such as peels, pulp and seeds have intensified in recent years, there are limited efforts on the valorization of green leaves, which are agricultural field wastes. While studies in the literature progress on extracts obtained from such leaves, in this study, the juice of four different leaves was obtained by cold pressing without using any harsh chemicals or pretreatments, and the bioactivity and cytotoxicity of the resulting juices were examined. The findings showed that LJs, especially beetroot LJ, have undeniable bioactivity and cause significantly little or no cell death at appropriate concentrations. To diversify the direct consumption of the leaves as LJ and to increase the functionality of the beverage, water kefir fermentation was applied. Among all leaves, beetroot LJ was chosen due to the highest probiotic viability, sulforaphane content, and bioactivity. After 21 days of cold storage, fermentation continued slowly, and it was supported by the physicochemical changes, microbial counts and organic acid profile. Although untrained panelists gave higher scores, especially in the first week of storage, the overall acceptability remained on average, at the end of 21 days. This study sheds light on the valorization of agricultural wastes with an environmentally friendly approach, and takes the first step to use leaf juice for the production of upcycled food and/or food ingredients. Nevertheless, it is subject to certain limitations, including the variations in laboratory- and industrial-scale productions, the need for systematic collection of food waste for industrial-scale production, the variable composition of waste even depending on harvest time or soil, the small sample used in the sensory analysis, and the lack of direct correlation between *in vitro* and *in vivo* assays. Future studies may include the development of industrial-scale process designs or product formulations that expand its uses beyond beverages, such as for salad dressings. It would also be beneficial to examine the effects of consumption of fermented LJs on human health using *in vivo* models.

## Data Availability

The original contributions presented in the study are included in the article/Supplementary material, further inquiries can be directed to the corresponding author.
