# Supplementary material for: Exploring the valorization of green leaves accumulated as agricultural waste into plant-based fermented juices
Source: Front Nutr. 2026 Apr 21;13:1823095. doi: 10.3389/fnut.2026.1823095 (PMC13138924; doi:10.3389/fnut.2026.1823095)
Supplement: Supplementary file 1 [file Table_1.docx]

Supplementary Material

**Table S1.** Proximate composition of leaves.

| Leaf | Moisture  (%) | Protein  (mg/g leaf, DW) | Lipid  (mg/g leaf, DW) | Ash  (mg/g leaf, DW) |
| --- | --- | --- | --- | --- |
| Broccoli | 81.77±0.38^C^ | 243.53±29.67^A^ | 24.86±5.79^A^ | 108.27±10.12^B^ |
| Cauliflower | 87.65±0.36^A^ | 239.94±22.55^A^ | 24.29±4.16^A^ | 36.16±5.68^D^ |
| Beetroot | 85.50±0.11^B^ | 238.04±35.70^A^ | 20.30±5.90^A^ | 172.89±9.68^A^ |
| Black carrot | 66.60±0.90^D^ | 142.46±10.39^B^ | 6.80±0.72^B^ | 72.68±2.62^C^ |

Difference between values with different letters within the same column are statistically significant (*p*<0.05).
